# Reduced circulating regulatory T cells in primary Sjögren’s syndrome: the contribution of enhanced apoptosis and impaired survival

**DOI:** 10.3389/fimmu.2025.1603305

**Published:** 2025-08-22

**Authors:** Yanlin Wang, Yuhan Jia, Hui Guo, Min Feng, Yan Qin, Zhaojun Liang, Xiangcong Zhao, Chong Gao, Jing Luo

**Affiliations:** ^1^ Division of Rheumatology, Department of Medicine, The Second Hospital of Shanxi Medical University, Taiyuan, Shanxi, China; ^2^ Shanxi Key Laboratory of Rheumatism Immune Microecology, The Second Hospital of Shanxi Medical University, Taiyuan, Shanxi, China; ^3^ Shanxi Precision Medical Engineering Research Center for Rheumatology, Shanxi, China, The Second Hospital of Shanxi Medical University, Shanxi, China; ^4^ Second Clinical Medical College, The Shanxi Medical University, Taiyuan, Shanxi, China; ^5^ Division of Nephrology, Department of Medicine, The Second Hospital of Shanxi Medical University, Taiyuan, Shanxi, China; ^6^ Division of Nephrology, Department of Medicine, Shenzhen Baoan Shiyan People’s Hospital, Shenzhen, Guangdong, China; ^7^ Department of Pathology, Brigham and Women’s Hospital, Harvard Medical School, Boston, MA, United States

**Keywords:** primary Sjögren’s syndrome, regulatory T cells, apoptosis, Caspase3, effector T cells (Teffs)

## Abstract

**Background:**

Regulatory T cells (Tregs) are found to be critical for maintaining immune tolerance to self-antigens; however, their status in primary Sjögren’s syndrome (pSS) remains unclear. We investigated alterations in the abundance of peripheral Tregs in a large pSS cohort and their implications for patients.

**Methods:**

Levels of CD4+CD25+FOXP3+Treg cells in the peripheral blood of 624 patients with pSS, and 93 healthy controls (HCs) were detected using modified flow cytometry (FCM). We then performed transcriptome sequencing of CD4+CD25+CD127-Treg cells, and used droplet digital PCR (ddPCR) to validate that the apoptosis-related genes were found in the sorted Treg cells. Apoptosis of CD4+CD25+CD127-Treg cells was verified using 7-AAD and annexin-V staining. We performed FOXP3/activated caspase-3 double immunohistochemistry to characterize features of the labial salivary glands.

**Results:**

The peripheral abundance of Treg cells from relapsing pSS patients was significantly contracted, especially in patients with high disease activity. We identified 187 upregulated and 674 downregulated DEGs in sorted Tregs from pSS patients, including five apoptotic pathway hub genes (XIAP, CASP3, CASP10, NFKBIA, and PMAIP1)-a finding consistent with increased Treg apoptosis in pSS. Active caspase-3 was detected in FOXP3+ cells within the minor labial salivary gland tissue of pSS patients. Higher levels of active Caspase-3 were correlated with lower Treg cell numbers. Interestingly, although the downregulation of the PI3K/AKT signaling pathway did not reach statistical significance, this vital pro-survival axis may still contribute to Treg impairment in pSS.

**Conclusions:**

These data suggest that the decreased peripheral abundance and increased apoptosis of Treg cells play an important role in the pathogenesis of pSS. Therefore, approaches for increasing Treg numbers *in vivo* could provide precise pSS therapy.

## 1 Introduction

Primary Sjögren’s syndrome (pSS) is a chronic systemic autoimmune condition characterized by a breakdown of self-tolerance. Histologically, extensive infiltration of lymphocytes is found in pSS target tissues, mainly in the salivary glands where CD4+T cells are predominant (1, 2). Excess activation of pro-inflammatory cells and mediators is conventionally considered to contribute to the formation of autoimmune diseases (1). Therefore, currently, pSS patients are required to take immunosuppressive drugs long-term, resulting in complications from global suppression of the immune response (3, 4).

Regulatory T cells (Tregs) are a specialized subset of T cells that act to suppress the immune response, thereby maintaining homeostasis and immune tolerance (5, 6). FOXP3, a master transcription factor, is the most specific marker of these cells and CD4+CD25+FOXP3+Treg cells have been extensively studied. In humans, CD127(low/-) could serve as surrogate markers for detecting and sorting living Tregs (7). Mutations in the FOXP3 gene lead to the development of dysfunctional Treg cells, resulting in severe autoimmunity and early-onset uncontrolled lymphoproliferation in mice (8). Additionally, Treg cells have been shown to be impaired in a variety of autoimmune diseases as evidenced by a reduction in Treg cell numbers, function, or survival (5, 7).

Treg cells normally constitute 1-5% of peripheral T cells. In a fully functional immune system, the size of the Treg peripheral abundance is maintained at a constant level by homeostatic mechanisms (9, 10). Reduced peripheral abundance of Treg cells is associated with numerous primary autoimmune diseases. However, there is no solid evidence regarding the peripheral abundance of Tregs in pSS patients. In this study, we used the absolute Treg count as a metric to assess their compartmentalized abundance in the peripheral circulation of this patient group. Furthermore, we performed a comprehensive investigation of changes in Treg cells in peripheral blood and salivary gland samples from pSS patients using flow cytometry, transcriptomic analyses, and immunohistology. Our findings provide further insight into the mechanisms underlying the progression of pSS and suggest that Caspase3 hub genes are potential therapeutic targets.

## 2 Materials and methods

### 2.1 Patients

**Figure 1:**
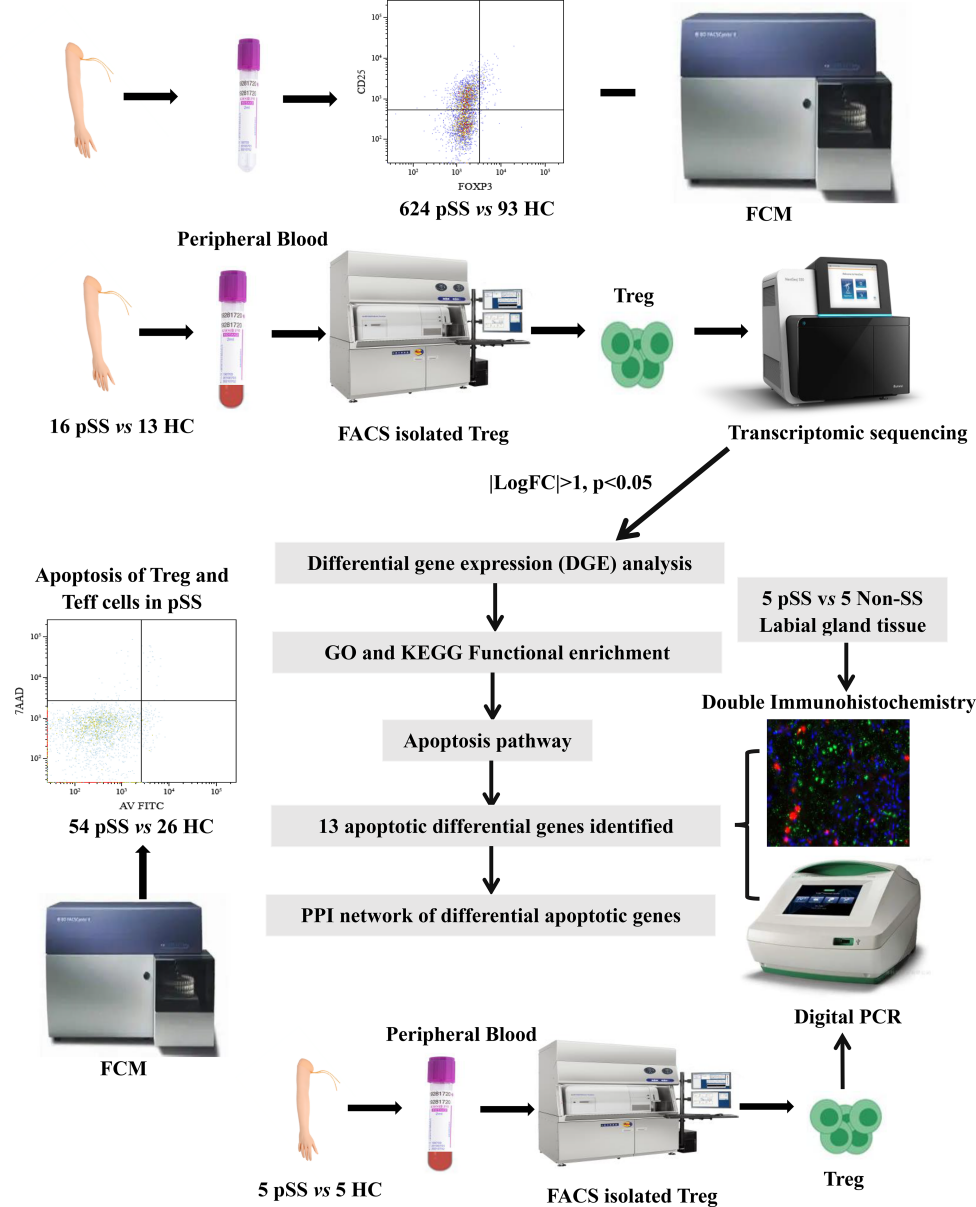
The flow diagram of the experiments in this study.

We enrolled 624 patients diagnosed with pSS from the Department of Rheumatology at the Second Hospital of Shanxi Medical University. Health controls (HCs) were from a health examination center (**Supplementary Table S1**). Of these 16 pSS patients and 13 HCs were recruited for RNA sequencing of Treg cells (**Supplementary Table S2**). In addition, 56 pSS patients and 24 matched HCs were enrolled in a prospective study to analyze apoptosis (**Supplementary Table S3**). Also, double immunofluorescence (IF) analysis was performed on 5 patients with pSS and 5 patients with non-SS sicca syndrome. Individuals presenting with clinically significant sicca symptoms (oral/ocular dryness) in the absence of inflammatory rheumatic disease were classified as having non-SS. Finally, the apoptosis genes of Treg cells from 5 pSS patients and 5 HCs were validated by droplet digital PCR (ddPCR). The entire research design is illustrated in **Figure 1**. All examinations were granted approval by the Medical Research Ethics Committee of the Second Affiliated Hospital of Shanxi Medical University (2024YX064), and written informed consent was obtained from all subjects.

### 2.2 Flow cytometry analysis and fluorescence-activated cell sorting (FACS) of Treg cells

We analyzed Th1 (CD4^+^IFN-γ^+^)/Th2 (CD4^+^IL-4^+^)/Th17 (CD4^+^IL-17^+^), and Treg (CD4^+^CD25^+^FOXP3^+^) cells as previously described (11). Peripheral blood mononuclear cells (PBMCs) were extracted from fresh peripheral blood. The PBMCs were immediately aliquoted into two parts for further use. Then 1,000 Tregs (CD4^+^CD25^hi^CD127^low^) were flow-sorted directly into lysis buffer using a FACSAria Fusion (BD Biosciences, San Jose, CA, USA)

### 2.3 RNA sequencing and data preprocessing

RNA was extracted from sorted Treg cells of HCs and pSS patients using the SMART-Seq v4 Ultra Low Input RNA Kit (Takara, Kyoto, Japan). cDNA was quantified using a Qubit 4.0 Fluorometer (Thermo Fisher Scientific). PCR products were then indexed using a Nextera XT DNA Library Prep Kit (Illumina, San Diego, USA). The libraries were pooled, quantified, and sequenced across 75 base pairs using a paired-end approach with a 75-cycle high-output flow cell on a NextSeq 550 (Illumina, San Diego, USA). The quality control of the reads was assessed with the FastQC tool, and two pSS PBMC samples were excluded after sequencing because of a high number of reads aligning to intronic regions, indicating genomic DNA contamination. The other samples passed the quality check. The depth ranged from 5 million to 7 million reads per sample. Raw counts were normalized using DESeq2 (Bioconductor). For all the samples,>90% of the reads were uniquely mapped to the human genome.

### 2.4 Differentially expressed genes and functional enrichment analyses

The princomp function in R was used to conduct a principal component analysis (PCA) and hierarchical clustering to visualize the characteristics of the pSS and HC groups. Subsequently, DEGs were identified based on the following criteria: adjusted p<0.05 and absolute value of log2 fold change |(FC) > 1|. All DEGs were visualized in volcano plots, and the top 50 DEGs were exhibited in a clustering heatmap.

Gene Ontology (GO) and Kyoto Encyclopedia of Genes and Genomes (KEGG) pathway analyses were conducted using the ClusterProfiler package. The bubble plot shows the KEGG and GO enrichment pathways, and the circle plot shows the functional characteristics of GO enrichment. The concentric circles from the outer to the inner layers represent: 1) Classification: (terms with identical colors belong to the same category) biological process (BP) = yellow, Cell composition (CC) = purple, molecular function (MF) = blue; 2) Total gene count per term (with color intensity reflecting the enrichment significance -log10(q)): the larger the value, the redder the color, and the greater the number of genes contained in each term; 3) the number of up-regulated genes in the term: the larger the value, the greater the number of differentially upregulated genes; 4) Rich factor: the number of upregulated genes in the term/the total number of genes in the term. The larger the RichFactor, the greater the degree of enrichment. The protein-protein interaction (PPI) networks of the DEGs were downloaded from the STRING database and structured by Cytoscape.

### 2.5 Apoptosis of effector T cells and Tregs in pSS

Snap-frozen PBMCs were stained with an Annexin V-FITC Kit (Miltenyi Biotec, Bergisch Gladbach, Germany). The PBMCs (1×10^6^) were incubated with BV510-anti-CD4, APC-anti-CD25 and PE-anti-CD127. Finally, the cells were resuspended with 100µL of 1X Annexin V Binding Buffer, and the apoptotic cell rate was analyzed using the BD FACSDiva software flow cytometer (BD FACSCanto II) within 1 hour. The samples were kept at 4°C away from light throughout the process.

### 2.6 Double immunofluorescence

Paraffin-embedded salivary gland tissues were sectioned into 3 μm sequential sections. The sections were then immunohistochemically stained with anti-human CD4 (Servicebio; GB11064-1), anti-FOXP3 (Servicebio; GB11093), and anti-Cleaved Caspase-3 (Servicebio; Q26 GB11532) to detect the expression of the corresponding target proteins in the tissue samples. For immunofluorescence, slides were incubated with the relevant secondary antibodies before 4’,6-diamidino-2-phenylindole (DAPI) nuclear counterstaining. Images were acquired with a Pannoramic MIDI scanner (3DHISTECH), and further analyzed using CaseViewer software. The number of FoxP3 and Caspase-3 double-positive cells was counted manually.

### 2.7 ddPCR

Total RNA of Treg cells was isolated using an RNeasy Mini Kit (QIAGEN, Hilden, Germany). The ddPCR master mix included 1× ddPCR Supermix for Probes (no dUTP, BIORAD), 0.9 μM primer and 0.25 μM probe (Applied Biosystems, Hilden, Germany) together with 2μL of cleaved sample DNA. The thermal cycling conditions included reverse transcription for 1 hour at 50°C, followed by 5 min at 95°C to activate the supermix. This was followed by 40 cycles of amplification at 95°C for 5 min (denaturation) and 53°C (BCL2, Caspase-3, PMAIP1)/59°C (DAXX, XIAP) for 40 sec (annealing), after which the supermix was deactivated with a stabilization at 72°C for 1 min, and then an infinite hold at 12°C. The cycled droplets were read individually with the QX200 Droplet Reader, and analyzed with the QuantaSoft droplet reader software (Bio-Rad).

### 2.8 Statistical analysis

Differences in gene expression with a corrected p-value<0.05 were considered statistically significant. Kruskal-Wallis H tests were used to analyze the differences between the pSS group and patient clusters. Fisher’s exact test was used to compare categorical variables. Statistical analyses and data visualization were performed using R, GraphPad Prism 8.0, and Cytoscape.

## 3 Results

### 3.1 The peripheral abundance of Treg cells decreased in pSS patients

**Figure 2:**
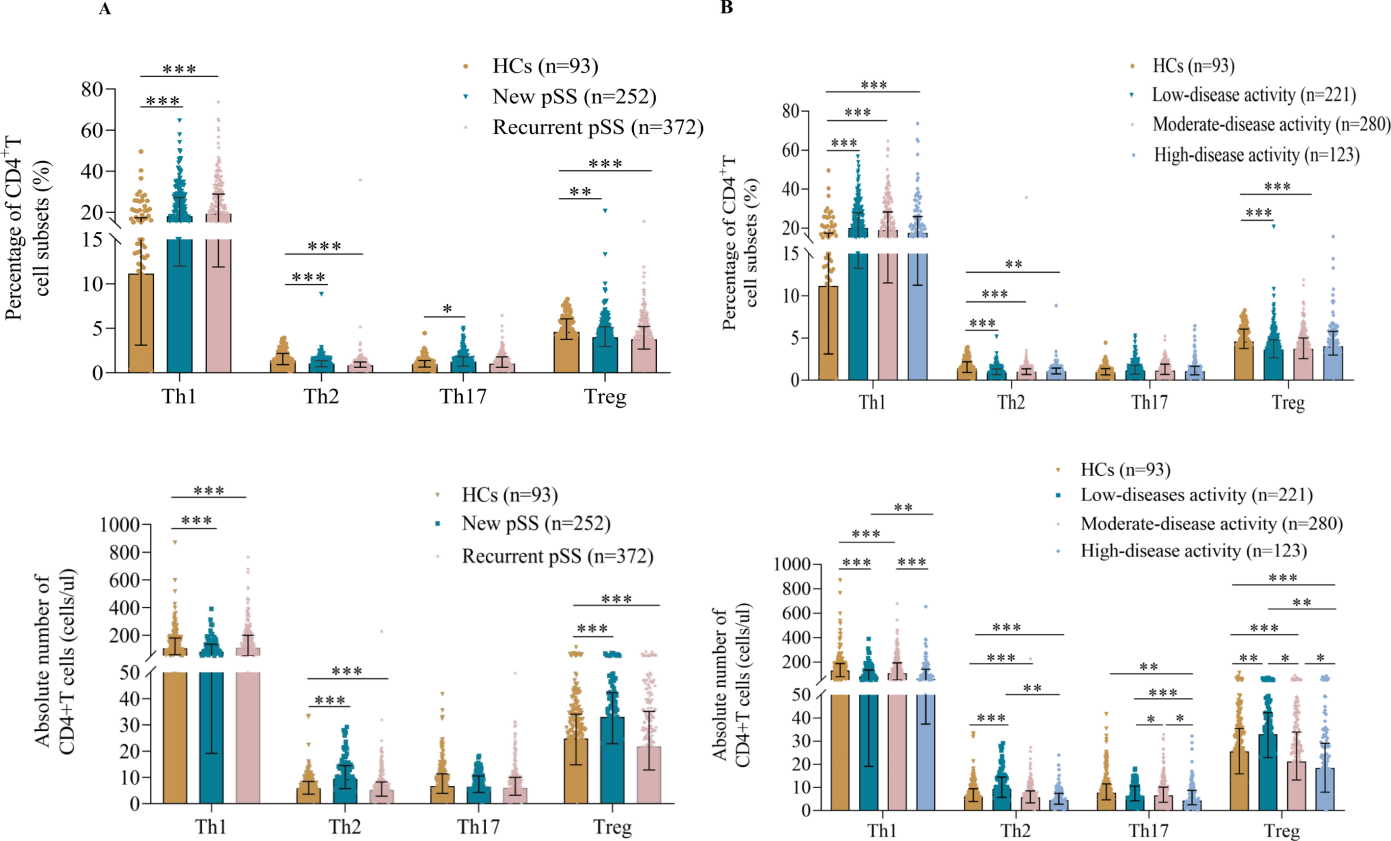
Treg cell levels were found to be reduced in pSS. **(A)** Pooled data showing the proportions and absolute numbers of CD4+T cell subsets in new and recurrent pSS patients. **(B)** Correlation of CD4+T cell subset levels with disease activity. All patients were divided into low (0≤ESSDAI<5, n=221), medium (5≤ESSDAI ≤ 14, n=280), or high (ESSDAI >14, n=123) disease activity groups. The data are presented as Median (Q25, Q75) and were analyzed using the Kruskal-Wallis H test. *p < 0.05, **p < 0.01, ***p < 0.001.

The demographic and clinical characteristics of these participants are shown in **Supplementary Table S1**. Interestingly, relapsed pSS patients had a significantly lower frequency and number of Treg and Th2 cells than HCs (**Figure 2A**). However, the percentage of Th17 cells in the new pSS patients was higher than in the HCs.

To explore the correlation between T cell subsets and disease activity (12), all pSS patients were divided into low (0≤ESSDAI<5), moderate (5≤ESSDAI ≤ 14) and high (ESSDAI>14) disease activity groups (**Figure 2B**). Notably, the lowest absolute numbers of Treg cells were found in patients with high disease activity, and the frequency of Treg cells was also significantly decreased in the low- and moderate-disease activity groups of pSS patients compared with controls. Additionally, the percentages of circulating Th1 cells in the low, moderate-, and high-disease activity groups were significantly higher than in the HCs. An opposite result was observed for Th2 cells, with lower percentages and numbers in different disease activity categories. However, the number of Th17 cells was significantly different only in patients with high-disease activity. Intriguingly, all CD4^+^T cell subsets in the high-disease activity group tended to decrease rather than increase, despite their role in immune function. 

### 3.2 Characterization of Treg cells by transcriptomic profiling

**Figure 3:**
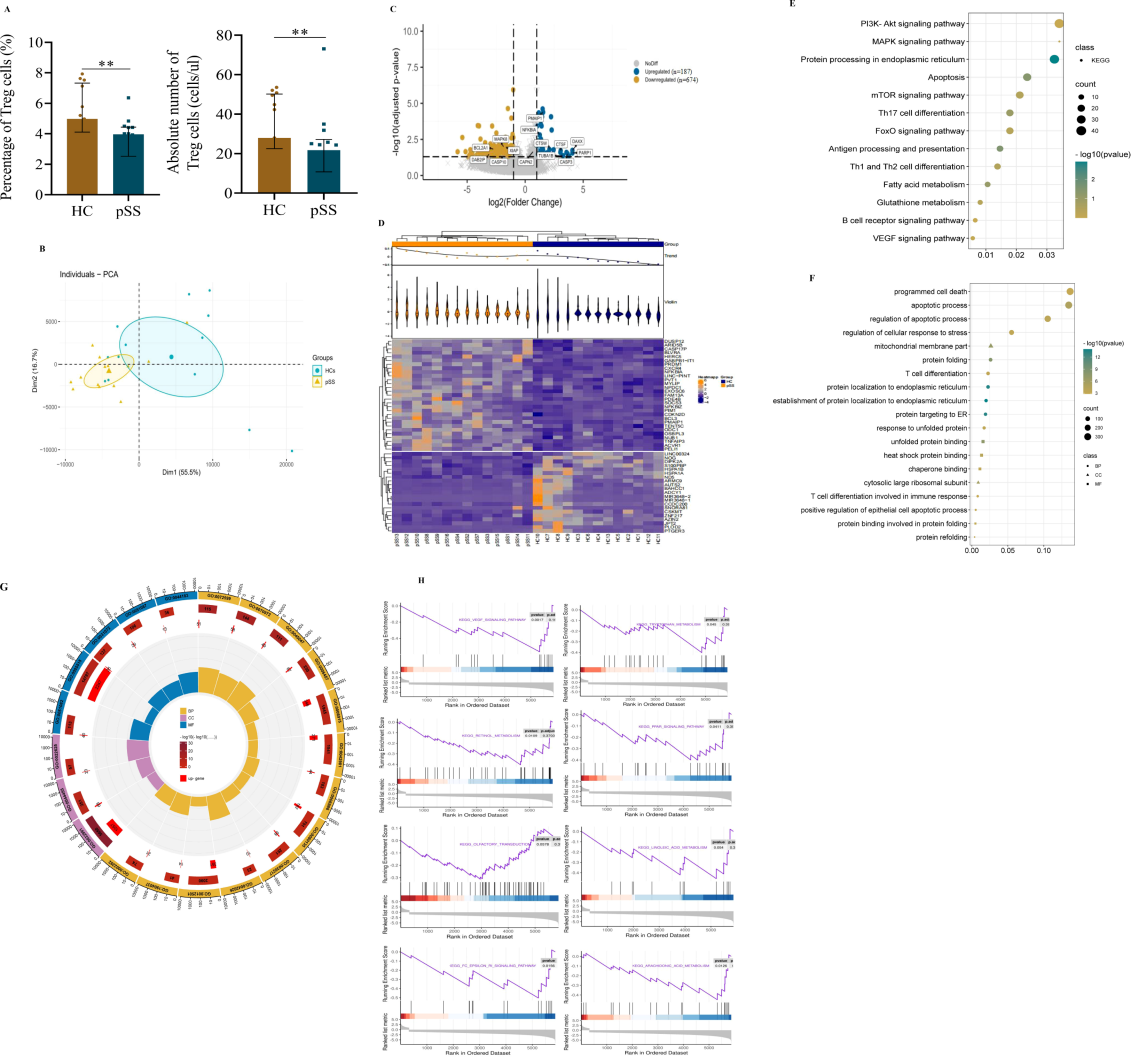
Transcriptome profiles of peripheral Treg cells in pSS patients and HCs. **(A)** Treg cell levels. **(B)** Principal component analysis of Treg cells. **(C)** Volcano plot of differentially expressed genes (DEGs) in Treg cells. Blue indicates upregulation(n=187); Yellow indicates downregulation (n=674). **(D)** Heatmap of the top 50 DEGs. **(E)** Bubble diagram of KEGG enrichment of Treg cell DEGs. **(F)** Bubble diagram of GO enrichment of Treg cell DEGs. **(G)** Circle map of GO enrichment of Treg cell DEGs. The concentric circles from the outer to the inner layers represent the following: 1) classification: (terms with identical colors belong to the same category) biological process (BP) = yellow, Cell composition (CC) = purple, molecular function (MF) = blue; 2) total gene count per term (with color intensity reflecting the enrichment significance -log10(q)): the larger the value, the redder the color, and the greater the number of genes contained in each term; 3) number of upregulated genes in the term: the larger the value, the greater the number of differentially upregulated genes; 4) rich factor: the number of upregulated genes in the term/the total number of genes in the term. The larger the Rich Factor, the greater the degree of enrichment. **(H)** Gene set enrichment analysis (GSEA) of DEGs in Treg cells in pSS and HC. Data were analyzed using an independent-samples T test. **p < 0.01.

For Treg analysis, we observed that the levels of CD4^+^CD25^+^CD127^-^ Treg cells in pSS patients were significantly lower than those in HCs (**Figure 3A**). RNA sequencing of circulating Tregs from pSS patients showed that the gene expression of the master regulator of the Treg pathway FOXP3, HELIOS (IKZF2), Interleukin-2 receptor alpha and beta chains (IL2RA and IL2RB), along with TNF receptor superfamily member 1B (TNFRSF1B) was relatively high in transcripts per million. Surprisingly, there were no significant differences in FOXP3, HELIOS, IL2RA, IL2RB, or TNFRSF1B expression in Treg cells between pSS patients and controls (**Supplementary Figure S2A**) (13, 14). This expression signature confirmed that the isolated cells were Treg cells and that there were no differences in the Treg gene signature between the two groups. 

Furthermore, we assessed the clustering of pSS and HCs which demonstrated that the two groups were robustly distinguished (**Figure 3B**). As shown in the volcano plot (**Figure 3C**), a total of 861 DEGs were identified, consisting of 187 up-regulated and 674 down-regulated genes. the heatmap showed distinct patterns between the two groups (**Figure 3D**). Moreover, the KEGG enrichment revealed that the majority of the related pathways included protein processing in the endoplasmic reticulum (ER), programmed cell death, and metabolism-related signaling pathways. Notably, the apoptosis pathway emerged as a focal point in the Treg cells of pSS patients (**Figure 3E**). Additionally, although the observed downregulation of the PI3K/AKT signaling pathway did not reach statistical significance (**Figure 3E**); this critical pro-survival axis may contribute to Treg impairment in pSS patients. This suggests that compromised Treg survival could represent a complementary mechanism underlying the reduced peripheral Treg frequency in patients, potentially working in concert with other established pathways of Treg deficiency (15).

Consistently, the regulation of the apoptotic process and protein localization to the ER were the top GO biological process entries (**Figures 3F, G**). Additionally, unfolded protein binding was the 10th enriched molecular function of DEGs (**Figure 3G**). To uncover the more nuanced mechanisms behind Treg abnormality and immune dysregulation, we conducted Gene set enrichment analysis. The GSEA of DEGs revealed the enrichment (p<0.05, p.adj>0.05) of genes involved in the VEGF signaling pathway, tryptophan metabolism, retinol metabolism, the PPAR signaling pathway, arachidonic acid metabolism, and the Fc epsilon RI signaling pathway in Treg cells of pSS patients. Nevertheless, there were no significant changes in olfactory transduction or linoleic acid metabolism (p>0.05) (**Figure 3H**).

### 3.3 Functional enrichment of apoptotic DEGs

**Figure 4:**
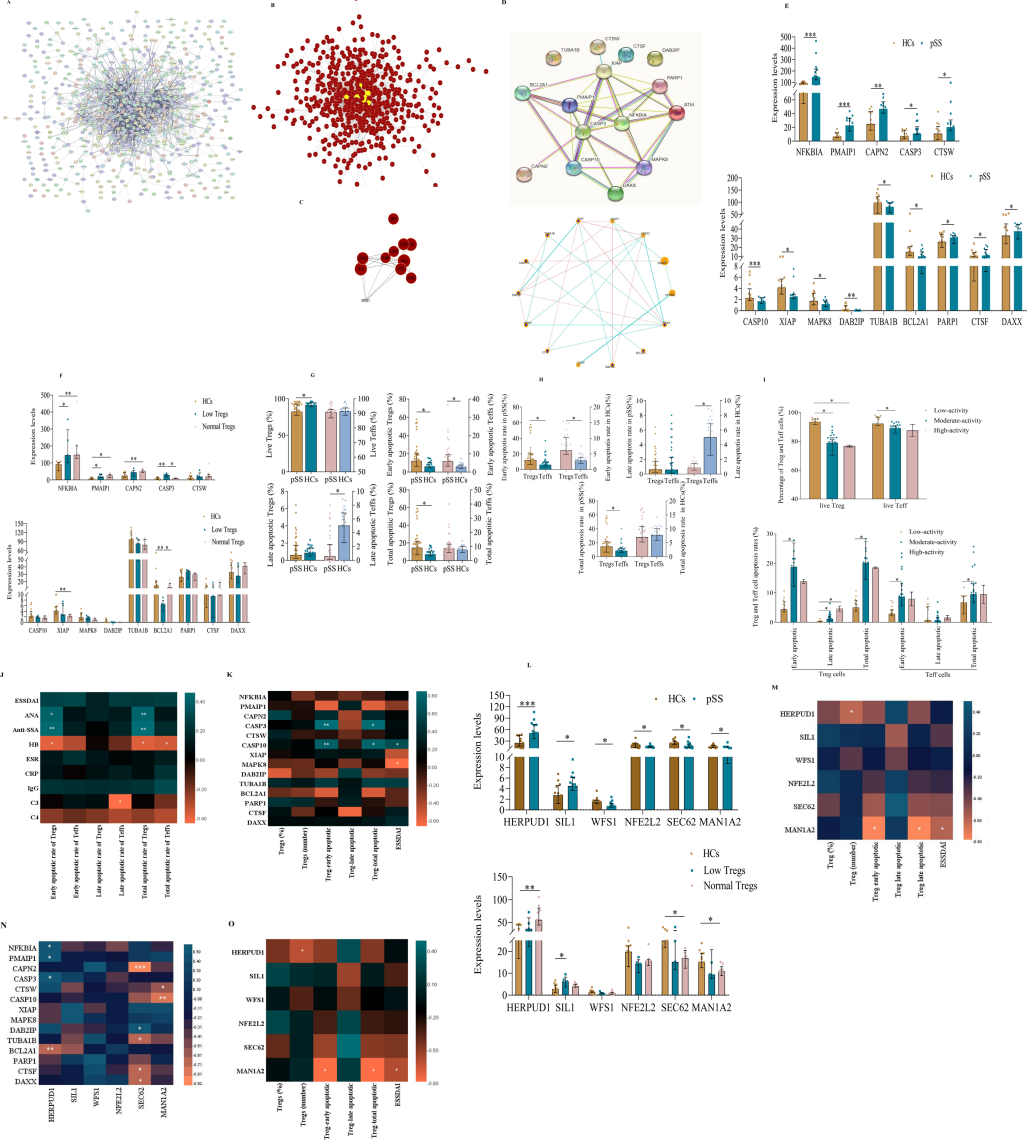
The apoptosis pathway is one of the most focused pathways in the peripheral Treg cells of pSS patients. **(A)** Construction of the PPI network based on the DEGs of Treg cells in the transcriptome sequencing data. **(B)** Construction of DEGs using the DEGREE algorithm in the Cytoscape software. **(C)** Core DEGs in the transcriptome sequencing of Treg cells. **(D)** Construction of the PPI network based on apoptosis-related DEGs of Treg cells derived from pSS and HCs. **(E)** Visualize the diagram of apoptosis-related DEGs in Treg cells from pSS patients and HCs. **(F)** Correlation between apoptosis-related DEGs and Treg cell counts. **(G-H)** Apoptosis of Tregs and effector T (Teff) cells in pSS and HCs. **(I)** Comparison of the rates of apoptosis in Tregs and Teff cells in the context of disease activity in pSS. **(J)** Associations between the different rates of apoptosis in Treg and Teff cells and pSS clinical features. **(K)** Correlation between the apoptotic DEGs of Treg cells and the different rates of apoptosis in Treg cells. **(L)** Plot diagram of endoplasmic reticulum stress DEGs between Treg cells from pSS and HCs. **(M)** Correlation between ER-related DEGs and Treg cell counts. **(N)** Correlation of apoptosis- and ER stress-related DEGs of Treg cells. **(O)** Correlation between ER stress-related DEGs in Treg cells and both Treg cell levels and apoptosis rate is significant. The data were analyzed using the independent-samples T test. *p < 0.05, **p < 0.01, ***p < 0.001.

To systematically analyze the relationships among all DEGs and apoptotic DEGs from transcriptomic profiling, we constructed separate PPI networks for each group (**Figures 4A, B** for all DEGs, **Figure 4D** for apoptotic DEGs). We found that all DEGs exhibit multifaceted interactions, notably with Caspase3 positioned as the network epicenter among all DEGs (**Figures 4A-C**). The PPI analysis also showed an interconnected network of 16 gene nodes and 24 edges, of which the five hub genes (XIAP, Caspase3, Caspase10, NFKB1A, and PMAIP1) participated in the apoptosis pathways (**Figure 4D**). Caspase3 was at the core position with a high degree of relatedness. Notably, to further interpret the relationship between decreased levels of Treg cells and apoptosis-related DEGs in pSS, 14 pSS patients were divided into low-level (n=5), and normal-level Tregs (unless otherwise stated, “normal-level Tregs” indicates quantitatively normal pSS Treg cells) groups (n=9). The highest levels of CASP3 were identified in patients with a low Treg count (**Figures 4E, F**). Conversely, there was a significant decrease in the expression of BCL2A1 in the low Treg group compared with normal Tregs or Tregs of HCs. However, no significant difference in other apoptotic DEGs was observed between the low and normal Treg groups.

### 3.4 Levels of apoptosis in Treg and Teff cells in pSS

To verify whether the decrease in the number of peripheral Tregs in pSS was caused by aberrant apoptosis, the levels of apoptotic Tregs and Teff cells were measured using Annexin V/7-AAD costaining (**Supplementary Figure S2B**) (16). The percentage of live Tregs (Annexin V-/7-AAD-) in pSS patients was significantly lower than that in controls, while the rates of early (Annexin V+/7-AAD-) and total apoptosis in Tregs in pSS patients were approximately twice as high as in HCs (**Figure 4G**). Moreover, the early apoptotic rate of Tregs was higher than that of Teff cells in both pSS and HCs. Also, the total apoptotic rate of Tregs was markedly higher than that of Teff cells in pSS but not in HCs (**Figure 4H**). We also analyzed the correlation between the rate of Treg apoptosis and disease activity (**Figure 4I**). As expected, the rate of early apoptotic Tregs in the low-activity group was significantly lower than that in the medium-activity group, and the percentage of late apoptotic Tregs in the low-activity group was also markedly lower than that in the medium-activity and high-activity groups. Moreover, the early and total apoptosis rates of Tregs were significantly correlated with the presence of ANA (r=0.419, p=0.011; r=0.430, p=0.009) and anti-SSA (r=0.451, p=0.005; r=0.471, p=0.003), but negatively correlated with HB (r=−0.372, p=0.018; r=−0.363, p=0.021), respectively. Additionally, late apoptotic Teff cells were negatively correlated with C3 (r=−0.422, p=0.045) (**Figure 4J**). For other indicators, the correlations were not significant. Strikingly, to investigate whether apoptotic DEGs correlated with the degree of apoptosis of Treg, we computed correlation heatmaps. Interestingly, we found that early apoptotic Tregs (r=0.867, p=0.005; r=0.767, p=0.027) and total apoptotic Treg cells (r=0.862, p=0.006; r=0.798, p=0.018) significantly correlated with CASP3 and CASP10 expression, respectively. In addition ESSDAI scores were positively correlated with CASP10 (r=0.647, p=0.017) expression and inversely correlated with MAPK8 expression(r=-0.639, p=0.019), respectively (**Figure 4K**). For other indicators, however, the correlations were not significant.

### 3.5 ER stress signaling may be related to Treg cell apoptosis in pSS

Noteworthy, a novel ER stress-mediated apoptosis pathway has recently emerged (17, 18). When ER stress occurs with high intensity, or it is prolonged, homeostasis is not restored and apoptosis is induced by ER-related molecules. As shown in **Figure 4L**, the expression of HERPUD1 and SIL1 of protein processing in ER DEGs in pSS Tregs was significantly higher than that of HC Tregs, while the expression of WFS1, NFE2L2, SEC62, and MAN1A2 was significantly lower. However, there was no difference observed in ER DEGs of HERPUD1, SIL1, WFS1, NFE2L2, SEC62, and MAN1A2 in the low and normal Treg groups (**Figure 4M**).

We also conducted a correlation analysis of Treg apoptosis and ERS-related genes in all pSS patients. We observed significantly positive correlations of HERPUD1 expression with NFKBIA, PMAIP1, and CASP3 expression, while inverse correlations were observed between HERPUD1 and BCL2A1. Furthermore, we found a significant correlation of SEC62 with DAB2IP and a significant inverse correlation of SEC62 expression with CAPN2, TUBA1B, CTSF, and DAXX. There was also a negative correlation of MAN1A2 with CTSW and CASP10 (**Figure 4N**). Correlations between protein processing in ER DEGs and the degree of apoptosis of Treg were also found with only MAN1A2 levels negatively correlated with the early apoptotic Tregs, total apoptotic Treg cells, and ESSDAI scores, but the correlation was weak (**Figure 4O**). Unexpectedly, the HERPUD1 expression was inversely correlated with the number of Treg cells. For other indicators, however, the correlations were not significant.

### 3.6 Active Caspase-3 was upregulated in Treg cells from pSS patients

**Figure 5:**
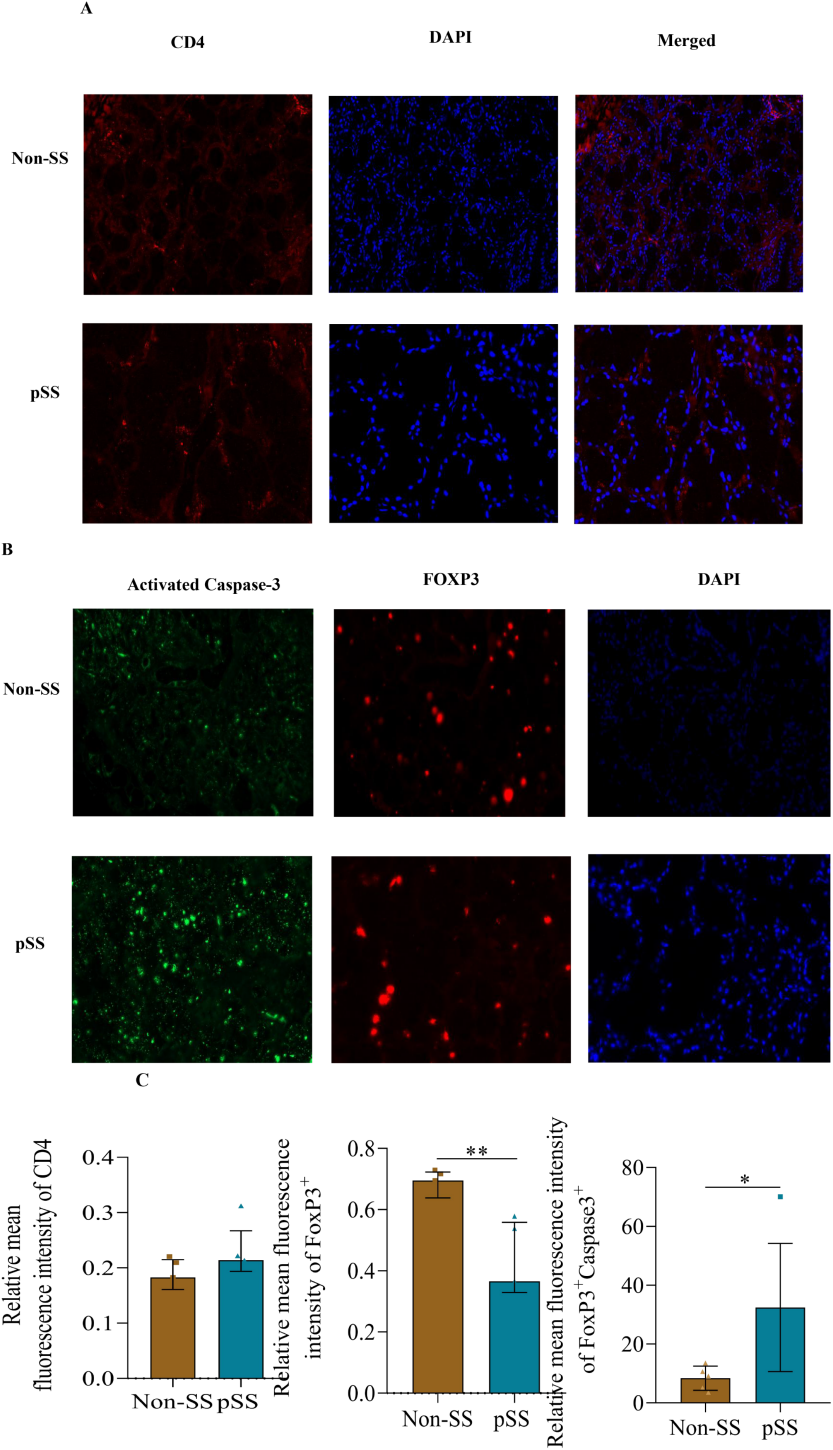
Active Caspase-3 (CASP3) was upregulated in Treg cells from pSS patients. **(A)** Representative immunohistological staining of CD4 (red), and DAPI (blue) of small labial glands from patients with pSS and healthy controls. **(B)** Representative immunohistological staining of Caspase3 (green), Foxp3 (red) and DAPI (blue) of small labial glands from patients with pSS and healthy controls. **(C)** Immunofluorescence quantification of Foxp3+Caspase3+ in small labial glands from patients with pSS and healthy controls. The data were analyzed using the independent samples t-test. *p < 0.05, **p < 0.01.

We focused on the active protein product of CASP3. Immunohistochemistry of SLG revealed that active Caspase 3 was highly expressed in pSS, compared with non-SS. Notably, SLG-infiltrating Foxp3+ cells exhibited a high level of active caspase 3 (**Figures 5B, C**), although there was no remarkable difference in CD4 expression (**Figures 5A, C**). Furthermore, the number of Foxp3+Treg cells in the salivary glands of SS patients was significantly lower than that in non-SS controls (**Figure 5C**).

Additionally, two upregulated genes (PMAIP1 and CASP3) and three downregulated genes (XIAP, BCL2A1, and DAXX) in pSS patients were selected for droplet digital PCR (ddPCR) verification (**Supplementary Figure S3A**). Compared to HCs, patients with pSS exhibited a higher expression of CASP3 and PMAIP13 levels, and lower BCL2A1, DAXX, and XIAP expression levels, although there were no significant differences in BCL2A1 and PAMIP1. Taken together, the expression trends of the five selected genes were consistent between the RNA-seq and ddPCR analyses, confirming the accuracy of our data.

## 4 Discussion

In our study, the peripheral abundance of Treg cells decreased in pSS patients. The reduced number and increased total apoptotic rates of Treg cells were associated with the increased expression of caspase 3 in these cells. Therefore, we provide the first evidence of a quantity defect of Treg cells associated with the pathogenesis of pSS, which is a potential target for precision medicine in patients with pSS and a reduced pool of Treg cells.

Early studies have used different markers to detect Tregs, resulting in conflicting conclusions (7, 19). Since CD4^+^CD25^+^FOXP3^+^ T cells have been widely studied as Tregs, we also measured their levels in this study. (9, 20). The results show that the absolute number of peripheral Tregs was significantly lower in pSS patients, especially those with high disease activity. Furthermore, the number of CD4^+^Foxp3^+^Treg cells in the salivary glands of pSS patients was distinctly lower than that in non-SS controls. These findings are in accordance with those of Li X et al., who reported a remarkable reduction in CD25^+^ or FOXP3^+^ T cells in pSS salivary glands (21). The above findings of decreased numbers of Treg cells in both peripheral and salivary glands indicate a decrease in Treg peripheral abundance in pSS patients. Notably, we also found that the absolute Treg cell count exhibits a stronger inverse correlation with disease severity, and the absolute number of anti-inflammatory Tregs was six times greater than that of pro-inflammatory cells in the peripheral blood of HCs; an increase in effector T cells and/or a decrease in Treg cells may disrupt the balance between pro- and anti-inflammatory cells. Additionally, the percentages of circulating Th1 cells in the low-, moderate-, and high- disease activity groups were significantly higher compared to the HCs. However, while Th1 cells contribute to IFN-γ production, their effects are largely mediated through B-cell activation rather than direct tissue damage (22). In contrast, Tregs play a direct role in controlling the truly pathogenic Th17 population (via IL-10 and TGF-β), with their functional impairment strongly correlating with glandular destruction in clinical biopsies (23). Furthermore, Treg dysfunction (characterized by reduced Foxp3 expression and impaired suppressive capacity) represents a fundamental defect in pSS, as demonstrated in multiple animal models (24, 25). Thus, these observations suggest that the immune imbalance associated with the development of pSS is due to an absolute decrease in Tregs but not an increase in Teff cells, as is generally believed; at least one of the causes of immune tolerance breaking is the decreased peripheral abundance of Tregs. 

Furthermore, our finding that Treg peripheral abundance is reduced challenges the conventional immunosuppressive therapy, which inhibits all cells, including Treg cells (26). Therefore, we propose immunomodulation therapy to increase Treg cell peripheral abundance and/or enhance the suppressive activity of Treg cells for patients with low Treg cell counts, such as those treated with low-dose IL-2 and an mTOR inhibitor (27, 28). However, studies on the heterogeneity of Treg cell subsets have revealed distinct subpopulations with different functions in controlling the immune response and inducing peripheral tolerance (29). Furthermore, pSS is a complex and heterogeneous condition and the Treg pool in pSS patients should also be thoroughly studied (30, 31).

Various potential factors may affect the peripheral abundance of Tregs in pSS patients, including reduced differentiation and proliferation of Tregs from naïve T cells and other progenitors, along with increased susceptibility to apoptosis (32–34). Apoptosis, a delicate process, is a predominant mechanism for maintaining peripheral lymphocyte homeostasis; however, it is not always good (35, 36). Thus, immune disorders, such as immunodeficiency and autoimmunity, may emerge from the dysregulation of lymphocyte apoptosis. However, apoptosis in pSS Tregs remains unclear. Our RNA-seq analysis identified numerous DEGs associated with apoptosis in Treg cells from pSS patients. Furthermore, the regulation of the apoptotic process and the localization of proteins to the ER were the top GO biological process entries. Importantly, the total apoptotic rate of Tregs increased greatly in pSS patients, while there was no significant change in Teff cells. Moreover, the increased apoptosis rate of Tregs was in line with disease activity. Notably, active caspase-3 levels were higher in CD4^+^Foxp3^+^ cells of LSG from pSS patients than in those from non-pSS patients. In addition to increased apoptosis, given the well-established role of PI3K/AKT as a pro-survival axis in lymphocytes (37), this observation suggests that impaired Treg survival mediated by deficient PI3K/AKT activation may represent an additional mechanism driving the reduced peripheral Treg frequency in pSS. While apoptosis directly reduces Treg numbers, suppressed PI3K/AKT signaling could further diminish the Treg pool by compromising long-term cellular viability, homeostatic maintenance, or resistance to extrinsic stressors. This dual defect in both survival and apoptotic pathways may synergistically exacerbate Treg deficiency in pSS, potentially contributing to disease progression.

In addition, the GSEA of DEGs revealed the enrichment of genes involved in the VEGF signaling pathway, tryptophan metabolism, retinol metabolism, the PPAR signaling pathway, arachidonic acid metabolism, and the Fc epsilon RI signaling pathway in Treg cells of pSS patients. These results portray the multifaceted functional derangements in pSS Treg cells, which are characterized by a compromised immunoregulatory capacity, dysregulated inflammatory responsiveness, aberrant pro-angiogenic signaling, and impaired metabolic fitness (38). This concerted dysregulation across pivotal cellular processes identifies salient candidate pathways implicated in the immune dysfunction intrinsic to pSS pathobiology. However, confirmatory investigations in substantially larger and more diverse patient cohorts are imperative to corroborate these observations.

Caspase 3 acts as an executioner in caspase-mediated apoptosis, and its expression positively correlates with the rate of apoptosis in cells (39, 40). Indeed, we observed that the higher expression of CASP3 positively correlates with the increased early apoptotic Tregs and total apoptotic Treg cells. And ddPCR showed that pSS patients exhibited higher CASP3 expression. Furthermore, CASP3 expression was significantly higher in patients with low-Treg levels than in either patients with normal-Treg levels or HCs. Conversely, in the BCL2 family, BCL2A1 has an antiapoptotic effect (41, 42). There was a significant decrease in BCL2A1 expression in pSS patients with low levels of Tregs. Additionally, pSS patients exhibited higher BCL2A1 expression levels than HCs, although no significant difference was observed. Therefore, we speculate that increased apoptosis of Treg cells plays a crucial role in the pathogenesis of pSS by inducing apoptosis signaling via upregulation of Caspase3 expression. In conclusion, we suggest that increased Treg cell apoptosis is one cause of decreased peripheral abundance. 

The ER is an indispensable and elaborate eukaryotic organelle; the unfolded protein response (UPR) assists in protein synthesis by reducing misfolded or unfolded proteins (17, 43). However, if the UPR fails to manage these proteins, cellular apoptosis pathways are triggered. Notably, several studies have suggested that ER stress (ERS) is involved in cell apoptosis (44, 45). Particularly, HERPUD1 is an important early marker of ERS and is involved in the ubiquitination and degradation of several unfolded proteins, and HERPUD1 has a dual effect on the apoptosis of different cells (46). Lin et al. found that microRNA-384-mediated upregulation of HERPUD1 promotes endothelial cell apoptosis induced by angiotensin II (47). However, HERPUD1 promotes cell survival under endoplasmic reticulum stress conditions by inhibiting apoptosis in neurons and glioma cells (48). Additionally, MAN1A2 orchestrates apoptosis through the modulation of signaling pathways (primarily TGF-β and ER stress) by regulating the N-glycosylation status of critical receptors and signaling proteins (49). We found significant positive correlations between HERPUD1 expression and NFKBIA, PMAIP1, and CASP3 expression, while inverse correlations were found between HERPUD1 and BCL2A1. There were also negative correlations between MAN1A2 expression and CTSW and CASP10. Furthermore, MAN1A2 levels were negatively correlated with the early apoptotic Tregs, total apoptotic Treg cells, and ESSDAI scores. Thus, we propose that in the context of Treg cells in pSS patients, sustained ER stress could lead to prolonged HERPUD1 upregulation. This upregulation could significantly increase the expression of cleaved caspase-3, the most important terminal cleavage enzyme in apoptosis, thereby enhancing pro-apoptotic signaling. Similarly, MAN1A2, which is involved in N-glycan processing, could influence Treg cell survival by affecting glycoprotein folding and ER quality control. Dysregulation of MAN1A2 may exacerbate ER stress and contribute to apoptotic pathways. The specific mechanisms by which HERPUD1 and MAN1A2 are involved in regulating apoptosis need to be further studied in pSS Treg cells.

## 5 Conclusion

In summary, this study is the first comprehensive investigation of changes in the peripheral abundance of Treg cells and in the transcriptome profile of Treg cells in pSS patients. The peripheral abundance of Treg cells decreased in pSS, which was linked to impaired survival and enhanced susceptibility to apoptosis, with the increased apoptosis likely driven by the dysregulation of the apoptotic signaling pathway and the ER stress-mediated mechanism. These data strongly suggest that increasing the peripheral abundance of Treg cells and decreasing their apoptosis should be a potential therapy for pSS.

## Data Availability

The original contributions presented in the study are included in the article/**Supplementary Material**. Further inquiries can be directed to the corresponding author.

## Supplementary material

Supplementary Table 1 Clinical and laboratory features of patients with pSS studied for immune subtype analysis. *pSS, primary Sjögren’s Syndrome; HCs, healthy controls; ESSDAI, European League Against Rheumatism Sjögren’s Syndrome Disease Activity Index; WBC, white blood cell; RBC, red blood cell; HB, Hemoglobin; PLT, Platelet; LYMP, lymphocyte; NEUT, neutrophil; ESR, erythrocyte sedimentation rate; CRP, C-reactive protein; IgG, immunoglobulin G; C3, complement 3; C4, complement 4; ANA, antinuclear antibody; anti-SSA, anti-Sjögren’s syndrome-related antigen A; RF, rheumatoid factor. *P ≤* 0.05 was considered statistically significant. ^a^The focus score indicates the number of inflammatory foci containing more than 50 mononuclear cells per 4 mm^2^ biopsy.

Supplementary Table 2 Clinical and laboratory features of patients with pSS studied for RNA sequencing.

Supplementary Table 3 Clinical and laboratory features of patients with pSS studied for apoptosis analysis

Supplementary Figure 1 The laboratory characteristics of the apoptosis list in the control and the pSS group.

Supplementary Figure 2 Gene expression of the master regulator of the Treg cells. **(A)** Plot diagram showing the expression levels of the five Treg cell master regulators (FOXP3, HELIOS, IL2RA, IL2RB, and TNFRSF1B) in healthy controls (HCs) (n=5) and patients with pSS (n=5) based on transcriptome sequencing. The data were analyzed using the Mann-Whitney U test. P<0.05 indicates statistical significance. **(B)** Representative flow cytometry analysis of Treg and Teff cell apoptosis in the peripheral blood of pSS patients and HCs.

Supplementary Figure 3 Apoptosis-related DEGs in Treg cells. **(A)** ddPCR validated the five apoptosis-related DEGs in Treg cells from subjects. **(B)** We analyzed expression levels of five apoptosis-related genes in Treg cells from pSS patients and HCs using ddPCR.
