# Supplementary material for: Reduced circulating regulatory T cells in primary Sjögren’s syndrome: the contribution of enhanced apoptosis and impaired survival
Source: Front Immunol. 2025 Aug 22;16:1603305. doi: 10.3389/fimmu.2025.1603305 (PMC12411454; doi:10.3389/fimmu.2025.1603305)
Supplement: Supplementary Table 1 — Clinical and laboratory features of patients with pSS studied for immune subtype analysis. *pSS, primary Sjögren’s Syndrome; HCs, healthy controls; ESSDAI, European League Against Rheumatism Sjögren’s Syndrome Disease Activity Index; WBC, white blood cell; RBC, red blood cell; HB, Hemoglobin; PLT, Platelet; LYMP, lymphocyte; NEUT, neutrophil; ESR, erythrocyte sedimentation rate; CRP, C-reactive protein; IgG, immunoglobulin G; C3, complement 3; C4, complement 4; ANA, antinuclear antibody; anti-SSA, anti-Sjögren’s syndrome-related antigen A; RF, rheumatoid factor. P ≤ 0.05 was considered statistically significant. aThe focus score indicates the number of inflammatory foci containing more than 50 mononuclear cells per 4 mm2 biopsy. [file DataSheet1.docx]

**Supplementary materials**

**Table S1 Clinical and laboratory features of patients with pSS studied for immune subtype analysis**

|  | Whole blood | | |
| --- | --- | --- | --- |
|  | HCs (n = 93) | pSS (n = 624) | *P* |
| Age, mean±SD years | 51.29±12.77 | 56.00±14.05 | 0.585 |
| No. of female patients/no. of male patients | 88/5 | 590/34 | 0.977 |
| ESSDAI score | - | 8.75±6.82 | - |
| WBC, 10^9^/L | 5.53±1.25 | 5.56±2.93 | 0.906 |
| RBC, 10^9^/L | 4.81±0.45 | 4.14±1.01 | <0.001* |
| HB, g/L | 147.47±1.48 | 144.06±2.69 | 0.596 |
| PLT, 10^9^/L | 236.63±51.71 | 200.32±93.96 | <0.001* |
| LYMP, 10^9^/L | 1.85±0.53 | 1.52±0.72 | 0.013* |
| NEUT, 10^9^/L | 3.16±0.90 | 3.59±4.33 | 0.304 |
| ESR, mm/h | 10.91±0.67 | 38.67±1.40 | <0.001* |
| CRP, mg/L | 3.07±0.31 | 11.65±1.08 | 0.013* |
| IgG, g/L | 12.45±2.53 | 15.76±7.41 | 0.001* |
| IgA, g/L | 2.46±1.10 | 3.10±2.04 | 0.021* |
| IgM, g/L | 1.04±0.46 | 1.28±0.89 | 0.055 |
| Serum C3, g/L | - | 0.83±0.23 | - |
| Serum C4, g/L | - | 0.19±0.09 | - |
| ANA positive, no. (%) | - | 372/545 (68.26) | - |
| Anti-SSA positive, no. (%) | - | 245/536 (45.71) | - |
| Anti-SSB positive, no. (%) | - | 93/536 (17.35) | - |
| Anti-Ro52 positive, no. (%) | - | 276/536 (51.49) | - |
| RF positive, no. (%) | - | 162/526 (30.80) | - |
| Parotid and submandibular  gland enlargement, no. (%) | - | 58 (9.29) | - |
| Extraglandular involvement, no. (%) | - | 134 (21.47) | - |
| Xerostomia no. (%) | - | 548 (87.82) | - |
| Keratoconjunctivitis sicca no. (%) | - | 421 (67.47) | - |
| Dental ulcer no. (%) |  | 90 (14.42) |  |
| Focus score^a^ ≥ 1, no. (%) | - | 349/437 (79.86) | - |

*pSS = primary Sjögren’s Syndrome; HCs = healthy controls; ESSDAI = European League Against Rheumatism Sjögren’s Syndrome Disease Activity Index; WBC = white blood cell; RBC = red blood cell; HB = Hemoglobin; PLT = Platelet; LYMP = lymphocyte; NEUT = neutrophil; ESR = erythrocyte sedimentation rate; CRP = C-reactive protein; IgG = immunoglobulin G; C3 = complement 3; C4 = complement 4; ANA = antinuclear antibody; anti-SSA = anti-Sjögren’s syndrome-related antigen A; RF = rheumatoid factor. *P*≤0.05 was considered statistically significant. ^a^The focus score indicates the number of inflammatory foci containing more than 50 mononuclear cells per 4 mm^2^ biopsy.

**Table S2 Clinical and laboratory features of patients with pSS studied for RNA sequencing**

|  | RNA sequencing | | |
| --- | --- | --- | --- |
|  | HCs (n = 13) | pSS (n = 16) | *P* |
| Age, mean±SD years | 57.77±10.24 | 61.80±11.21 | 0.316 |
| No. of female patients/no. of male patients | 11/2 | 15/1 | 0.573 |
| ESSDAI score | - | 11.00 (5.00, 17.25) | - |
| WBC, 10^9^/L | 5.45 (4.60, 6.53) | 3.69 (3.15, 6.24) | 0.156 |
| RBC, 10^9^/L | 4.62 (4.45, 5.29) | 3.82 (3.07, 4.17) | <0.001* |
| HB, g/L | 147.00 (135.00, 166.50) | 115.50 (90.00, 131.75) | <0.001* |
| PLT, 10^9^/L | 241.00 (229.00, 279.00) | 230.00 (155.75, 279.50) | 0.618 |
| LYMP, 10^9^/L | 1.87 (1.55, 2.17) | 1.26 (0.89, 1.60) | 0.013* |
| NEUT, 10^9^/L | 3.11 (2.42, 4.13) | 2.30 (1.68, 4.01) | 0.294 |
| ESR, mm/h | 13.00 (5.50, 15.00) | 47.00 (32.00, 110.25) | 0.001* |
| CRP, mg/L | 11.20 (9.91, 13.15) | 11.24 (0.00, 36.90) | 0.786 |
| IgG, g/L | 11.20 (9.91, 13.15) | 14.55 (10.85, 24.08) | 0.026* |
| IgA, g/L | 1.81 (0.97, 2.81) | 3.59 (2.36, 4.98) | 0.019* |
| IgM, g/L | 0.94 (0.60, 1.18) | 1.19 (0.76, 1.51) | 0.204 |
| ANA positive, no. (%) | - | 10/12 (83.33) | - |
| Anti-SSA positive, no. (%) | - | 9/13 (69.23) | - |
| Anti-SSB positive, no. (%) | - | 4/13 (30.77) | - |
| Anti-Ro52 positive, no. (%) | - | 11/13 (84.62) | - |
| RF positive, no. (%) | - | 8/12 (66.67) | - |
| Parotid and submandibular  gland enlargement, no. (%) | - | 1 (6.25) | - |
| Extraglandular involvement, no. (%) | - | 3 (18.75) | - |
| Xerostomia no. (%) | - | 15 (93.75) | - |
| Keratoconjunctivitis sicca no. (%) | - | 10 (62.50) | - |
| Dental ulcer no. (%) | - | 3 (18.75) | - |
| Focus score^a^ ≥ 1, no. (%) | - | 5/5 (100) | - |

**Table S3 Clinical and laboratory features of patients with pSS studied for apoptosis analysis**

|  | Apoptosis | | |
| --- | --- | --- | --- |
|  | HCs (n = 26) | pSS (n = 54) | *P* |
| Age, mean±SD years | 57.32±8.90 | 57.85±12.36 | 0.688 |
| No. of female patients/no. of male patients | 26/0 | 52/2 | 0.723 |
| ESSDAI score | - | 6.00 (2.00, 8.00) | - |
| WBC, 10^9^/L | 5.04 (4.47, 6.59) | 5.02 (3.86, 6.50) | 0.509 |
| RBC, 10^9^/L | 4.40 (4.24, 4.76) | 4.22 (3.80,4.49) | 0.005* |
| HB, g/L | 136.00 (129.00, 144.25) | 129.00 (118.00, 137.00) | 0.035* |
| PLT, 10^9^/L | 265.00 (233.75, 290.75) | 175.00 (149.00, 214.00) | <0.001* |
| LYMP, 10^9^/L | 1.82 (1.58, 2.14) | 1.44 (0.93, 2.02) | 0.070 |
| NEUT, 10^9^/L | 2.85 (2.39, 4.01) | 2.81 (1.70, 3.64) | 0.489 |
| ESR, mm/h | 13.00 (6.75, 18.00) | 27.00 (15.00, 64.00) | <0.001* |
| CRP, mg/L | - | 2.92 (1.82, 3.92) |  |
| IgG, g/L | - | 13.50 (11.30, 21.70) |  |
| IgA, g/L | - | 3.56 (2.39, 4.57) |  |
| IgM, g/L | - | 1.17 (0.80, 2.22) |  |
| Serum C3, g/L | - | 0.85 (0.67, 0.94) | - |
| Serum C4, g/L | - | 0.19 (0.14, 0.23) | - |
| ANA positive, no. (%) | - | 38/50 (76.00) | - |
| Anti-SSA positive, no. (%) | - | 33/51 (64.71) | - |
| Anti-SSB positive, no. (%) | - | 12/51 (23.53) | - |
| Anti-Ro52 positive, no. (%) | - | 26/51 (50.98) | - |
| RF positive, no. (%) | - | 31 (57.41) | - |
| Parotid and submandibular  gland enlargement, no. (%) | - | 8 (14.81) | - |
| Extraglandular involvement, no. (%) | - | 32 (59.26) | - |
| Xerostomia no. (%) | - | 49 (90.74) | - |
| Keratoconjunctivitis sicca no. (%) | - | 38 (70.37) | - |
| Dental ulcer no. (%) | - | 5 (9.26) | - |
| Focus score^a^ ≥ 1, no. (%) | - | 29/32 (90.63) | - |

**FIGURE 1**

**FIGURE 1**

The laboratory characteristics of the apoptosis list in the control and the pSS group.

**FIGURE 2**

**A**

Teff cells


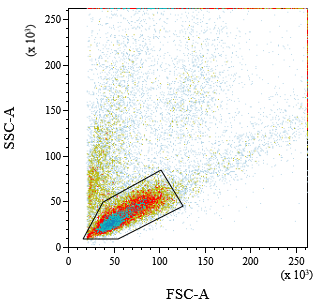

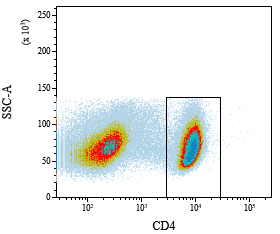

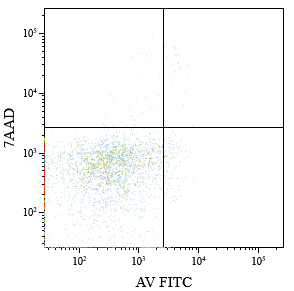

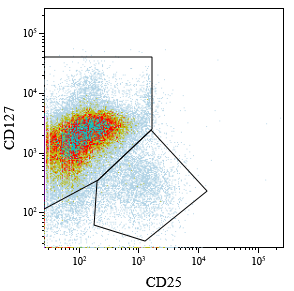

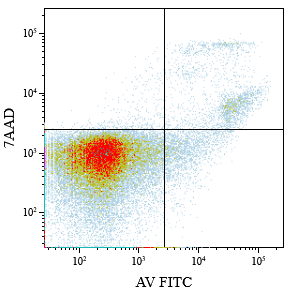


Treg

Teff

Live

Early apoptosis

Later apoptosis

Dead

Live

Early apoptosis

Later apoptosis

Dead

Treg cells

**B**

**FIGURE 2**

Gene expression of the master regulator of the Treg cells. (A) Plot diagram showing the expression levels of the five Treg cell master regulators (FOXP3, HELIOS, IL2RA, IL2RB, and TNFRSF1B) in healthy controls (HCs) (n=5) and patients with pSS (n=5) based on transcriptome sequencing. The data were analyzed using the Mann-Whitney U test. P<0.05 indicates statistical significance. (B) Representative flow cytometry analysis of Treg and Teff cell apoptosis in the peripheral blood of pSS patients and HCs.

**FIGURE 3**


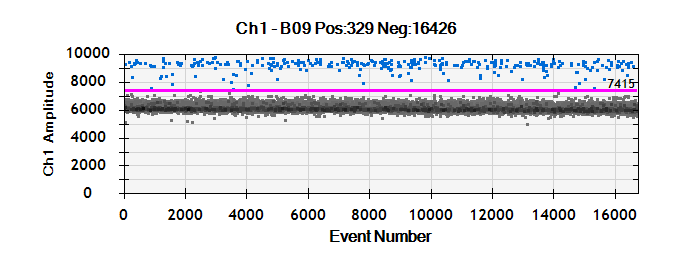


**XIAP**


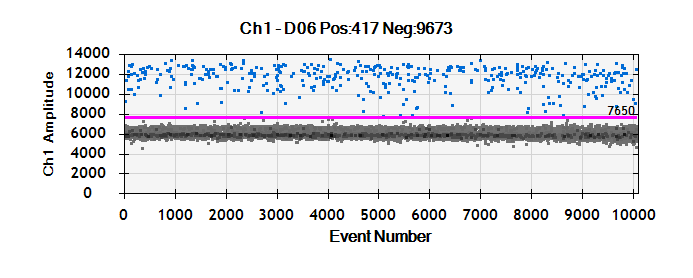


**PMAIP1**


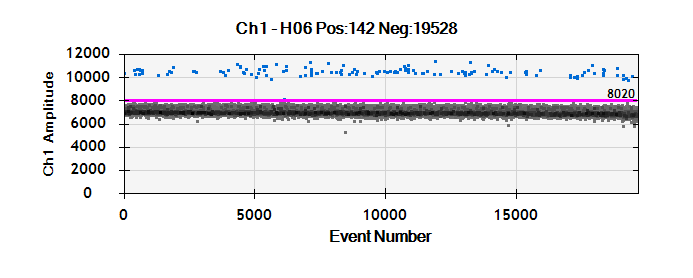


**CASP3**


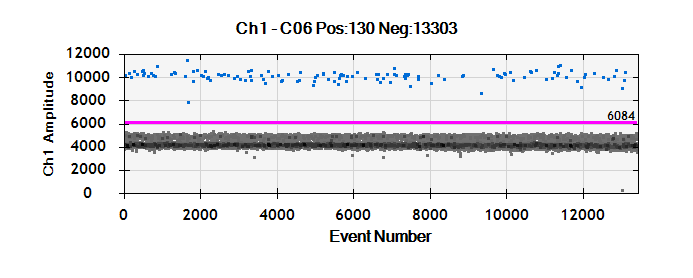


**BCL2A1**


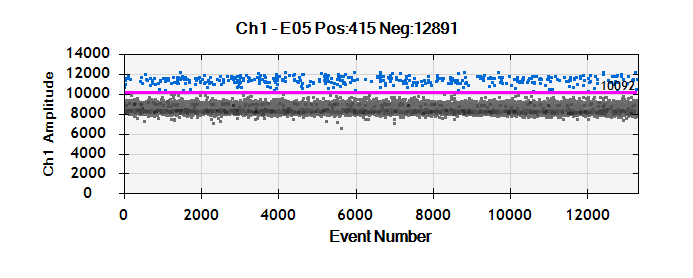


**DAXX**

**A**

**B**

**FIGURE 3**

Apoptosis-related DEGs in Treg cells. (A) ddPCR validated the five apoptosis-related DEGs in Treg cells from subjects. (B) We analyzed expression levels of five apoptosis-related genes in Treg cells from pSS patients and HCs using ddPCR.
